# COVID-19 Clinical Predictors in Patients Treated via a Telemedicine Platform in 2022

**DOI:** 10.3390/tropicalmed10080213

**Published:** 2025-07-29

**Authors:** Liliane de Fátima Antonio Oliveira, Lúcia Regina do Nascimento Brahim Paes, Luiz Claudio Ferreira, Gabriel Garcez de Araújo Souza, Guilherme Souza Weigert, Layla Lorena Bezerra de Almeida, Rafael Kenji Fonseca Hamada, Lyz Tavares de Sousa, Andreza Pain Marcelino, Cláudia Maria Valete

**Affiliations:** 1 Oswaldo Cruz Foundation (Fiocruz), Evandro Chagas National Institute of Infectious Diseases (INI), Clinical Epidemiology Laboratory, Rio de Janeiro 21040-900, Brazil; 2 Oswaldo Cruz Foundation (Fiocruz), Evandro Chagas National Institute of Infectious Diseases (INI), Leishmaniasis Clinical Research and Surveillance Laboratory, Rio de Janeiro 21040-360, Brazil; luciabrahim@gmail.com (L.R.d.N.B.P.); luiz.ferreira@ini.fiocruz.br (L.C.F.); cmvalete@gmail.com (C.M.V.); 3 Conexa Health, Rio de Janeiro 22040-002, Brazil; 4 Department of Otorhinolaryngology and Ophthalmology, School of Medicine, Federal University of Rio de Janeiro, Rio de Janeiro 21941-901, Brazil

**Keywords:** COVID-19, SARSCoV-2 virus, telemedicine

## Abstract

Coronavirus disease (COVID-19) is an infectious disease caused by the SARS-CoV-2 virus, whose 2020 outbreak was characterized as a pandemic by the World Health Organization. Restriction measures changed healthcare delivery, with telehealth providing a viable alternative throughout the pandemic. This study analyzed a telemedicine platform database with the goal of developing a diagnostic prediction model for COVID-19 patients. This is a longitudinal study of patients seen on the Conexa Saúde telemedicine platform in 2022. A multiple binary logistic regression model of controls (negative confirmation for COVID-19 or confirmation of other influenza-like illness) versus COVID-19 was developed to obtain an odds ratio (OR) and a 95% confidence interval (CI). In the final binary logistic regression model, six factors were considered significant: presence of rhinorrhea, ocular symptoms, abdominal pain, rhinosinusopathy, and wheezing/asthma and bronchospasm were more frequent in controls, thus indicating a greater chance of flu-like illnesses than COVID-19. The presence of tiredness and fatigue was three times more prevalent in COVID-19 cases (OR = 3.631; CI = 1.138–11.581; *p*-value = 0.029). Our findings suggest potential predictors associated with influenza-like illness and COVID-19 that may distinguish between these infections.

## 1. Introduction

COVID-19 is a viral illness caused by SARS-CoV-2 (Coronaviridae family). In March 2020, the World Health Organization characterized the COVID-19 outbreak as a pandemic [1]. The most common clinical manifestations in COVID-19 patients include fever or chills, fatigue, headache, muscle or body aches, dry cough, pneumonia, and dyspnea [2]. Comparison studies between COVID-19 cases and other common colds highlighted their different symptomatology, which can aid in the diagnosis of this disease [3,4,5].

The transmission dynamics of the disease since the onset of the pandemic have been closely associated with the emergence of multiple SARS-CoV-2 variants. The replacement of the Delta variant by Omicron began in December 2021, followed by a sharp increase in COVID-19 cases in January 2022 [6]. In Rio de Janeiro, Brazil, there was a notable rise in the incidence rate of COVID-19 cases in 2022 compared to the years 2021 and 2020, with 763,040; 310,398; and 223,025 confirmed cases, respectively. However, both the mortality and case fatality rates declined significantly [7]. This phenomenon is correlated with the emergence and sustained predominance of the Omicron variant in Brazil [8].

Infection caused by the Omicron variant has been associated with a milder clinical course and reduced severity in vaccinated individuals compared to infections caused by the Delta variant [9]. Differences in the clinical manifestations of COVID-19 have been highlighted following the introduction of Omicron. Wang [10] emphasizes that although the incidence of symptoms in patients infected with Omicron was lower than that observed with previous variants, the symptomatology tends to be more complex and requires differentiation from influenza.

During the pandemic, circulation-restricting and social distancing measures were recommended to decrease viral transmission in the population [1]. Early diagnosis allows immediate isolation to be indicated, decreasing the COVID-19 transmission time [11]. RT-qPCR is considered the gold-standard laboratory method for the diagnosis of SARS-CoV-2 [12]. The high specificity of RT-qPCR (i.e., the detection of nucleic acids in nasopharyngeal and oropharyngeal samples by reverse transcription quantitative polymerase chain reaction) rendered it the universal diagnostic method [2]. Rapid tests, or antigen tests, when performed within the first week of symptom onset, have a reported sensitivity of 83.8% [13]. Nevertheless, should this test be unavailable, a combination of clinical and laboratory features can be applied to aid in the diagnosis [3,5].

Restriction measures have changed healthcare delivery, with telehealth providing a viable alternative modality of care during the pandemic [14]. In 2020, elective outpatient care for stable patients was suspended in public health units in the state of Rio de Janeiro, Brazil, according to SES Resolution No. 2004/2020 of 18 March 2020 [15].

By definition, telemedicine encompasses the remote diagnosis and treatment of patients via telecommunication infrastructures, whereas telehealth encompasses any service used to provide healthcare remotely [16]. The characteristic pattern of patients using telehealth/telemedicine services is similar to that of other healthcare and digital health services [14]. Telehealth is comparable to in-person care for several clinical and process outcomes [14], and can be associated with cost savings for both patients and the broader healthcare sector [17]. A previous study using a dataset obtained from a state-level telehealth service demonstrated that the initial reports of symptoms via teleconsultation preceded the officially notified COVID-19 cases [18].

Telehealth services can serve as a valuable source of high-quality health data [18]. Although secondary health data are valuable for understanding health–disease processes, their quality depends on factors like data collection methods, standardization, sample size, and analytical techniques. Thus, clear quality criteria are essential to ensure reliable and valid information for public health research and decision-making [19,20].

Conexa Saúde [https://www.conexasaude.com.br (accessed on 3 July 2023)] is a telemedicine platform that connects patients and healthcare professionals through technological means. During the COVID-19 pandemic, the tool consolidated an extensive clinical database, used herein to evaluate telemedicine as a predictive diagnosis resource. Therefore, this study aims to examine retrospective data from a telemedicine platform to investigate predictors of COVID-19.

## 2. Materials and Methods

This is a retrospective study of patients seen via the Conexa Saúde telemedicine platform in 2022. The platform has a quality assurance and data security department. Professionals have access to the platform through passwords and digital signatures, and they sign a confidentiality and commitment agreement.

In this study, a pre-selection of the 3,394,978 consultations from Conexa was performed, resulting in a database of 88,287 records related to the study topic. The variable ‘patient report’ includes the patient’s description during medical care, similar to a medical record. Excel functions applied to this variable and the selection of the place of origin were used for data cleaning, resulting in 4600 records. Subsequently, all 4600 records were individually reviewed to exclude duplicates and extract data on signs and symptoms, diagnostic confirmation records, and the definition of cases and controls for the creation of new variables (Supplementary Figure S1).

A descriptive analysis of data quality demonstrated a high completeness of key variables: ‘date of care’, ‘care completion date’, ‘patient ID’, and ‘patient report’ were fully completed in 100% of records, while ‘sex’ and ‘age’ were completed in 94.6% and 99.8% of cases, respectively. The values for ‘sex’ and ‘age’ were within expected ranges. Data consistency and timeliness were confirmed through the variables ‘date of care’ and ‘care completion date’, which aligned with the study period. All duplicate entries were identified and removed using the ‘patient ID’ variable. All steps were validated by two independent researchers.

Inclusion criteria included patients whose data indicated residence in the state of Rio de Janeiro, Brazil, and confirmation of COVID-19 or influenza-like illness (i.e., due to other viral infections yielding similar symptoms). Exclusion criteria included duplicate cases, unconfirmed suspected cases, post-COVID-19 cases, diagnoses unrelated to influenza-like illness, and records with no description of signs or symptoms, only clinical confirmation cases. All records containing a medical description of a confirmed case with a positive test—regardless of whether the type of test was specified—were considered as cases. Controls were defined as all patients presenting with flu-like symptoms who tested negative for COVID-19 or had laboratory confirmation of another respiratory syndrome.

This study was approved by the Ethics and Research Committee of the Evandro Chagas National Institute of Infectious Diseases of the Oswaldo Cruz Foundation (Fiocruz), under opinion no. 5.001.681.

Analyses were conducted using Statistical Package for Social Sciences for Windows v. 16.0 (SPSS Inc., Chicago, IL, USA). Descriptive statistics were used to summarize the findings. The simple frequencies of the main signs and symptoms reported among cases confirmed as COVID-19 and controls were described. The association between categorical variables was verified by Pearson’s χ2 test of proportions, or Fisher’s exact test. *p*-values < 0.05 indicated statistically significant tests. Statistically significant variables were included in the logistic regression analysis. A binary logistic regression analysis was applied to develop the predictive model for the outcome of interest in this study. A multiple binary logistic regression model, controls v. COVID-19, was developed to obtain an odds ratio (OR) and a 95% confidence interval (CI). Backward elimination with the likelihood ratio test was applied to obtain statistically significant variables. The calibration of each final logistic model was evaluated via the Hosmer–Lemeshow goodness-of-fit test and a *p*-value > 0.05 indicated good agreement between the observed and predicted disease.

## 3. Results

Data from the telemedicine platform identified 4600 records from the state of Rio de Janeiro in 2022, of which 2052 (44.6%) were excluded: 390 duplicates, 396 suspected cases, 70 post-COVID-19 cases, 100 diagnoses not related to influenza-like illness, and 967 entries without reported data on signs and symptoms, and 129 cases with a clinical diagnosis without any report of laboratory confirmation. A total of 2548 patients were included, of which 2419 served as cases and 129 were controls. The majority of the patients included were women (1462; 60.6%), and there was no statistically significant difference in sex distribution between cases and controls (*p*-value = 0.182). The mean age of cases was 44.2 years (standard deviation: 14.8), which was significantly higher than the mean age of controls (40.8 years; standard deviation: 16.0), with this difference being statistically significant (*p*-value = 0.008) (Supplementary Table S1).

All confirmed cases had a medical record indicating laboratory confirmation for COVID-19. Of these, 1084 (44.8%) did not have the type of test specified, 960 (39.7%) were confirmed by RT-qPCR, and 375 (15.5%) were confirmed by antigen or rapid tests. Among controls, all individuals—except one who had a laboratory-confirmed H1N1 infection—had a medical report indicating a negative COVID-19 test result. Of the controls, 77 (44.8%) had no test type specified, 20 (15.5%) were tested by RT-qPCR, and 31 (24.0%) by antigen or rapid tests.

The presence of rhinorrhea, sneezing and burning nose, ocular symptoms, abdominal pain, rhinosinusopathy, and wheezing/asthma/bronchospasm were more frequent in controls, showing a negative association with COVID-19. Fatigue/tiredness showed a positive association with COVID-19 cases (Table 1).

Eight independent variables were entered into a multiple binary logistic regression analysis to identify independent predictors that distinguished COVID-19 from controls. Based on backward elimination, six factors were considered significant: rhinorrhea, ocular symptoms, abdominal pain, rhinosinusopathy, and wheezing/asthma and bronchospasm were more frequent in controls, with their presence indicating a greater probability of a flu-like illness than COVID-19. Tiredness and fatigue were 3 times more prevalent in COVID-19 cases and for every 10 years of age, the risk of having COVID-19 was 16% higher. The Hosmer–Lemeshow goodness-of-fit test showed a good fit of the model to the data (*p*-value = 0.762). The results of the binary logistic regression analysis are summarized in Table 2.

## 4. Discussion

Data from the telemedicine evaluation were used for the identification of factors that may aid in the early diagnosis of COVID-19 by differentiating it from other influenza-like illnesses. The differentiation is a challenge when the access to specific diagnostics for each virus is limited, due to the non-specific clinical characteristics of these diseases [5,21,22,23,24].

The use of telemedicine data is fundamental as it becomes increasingly ingrained in patient care provisioning. Previous studies have highlighted the high effectiveness of telehealth service data in anticipating new COVID-19 waves, which can both help medical professionals understand the dynamics of the epidemic and support the surveillance of different diseases [18,25,26].

We observed a predominance of women with COVID-19 in this study, consistent with findings reported in other studies [3,27,28]. We also found that with each additional year of age, the risk of COVID-19 infection increased. A meta-analysis conducted in 2021 reported that individuals aged 70 and older appear to have a 65% higher risk of COVID-19 infection [29].

In 2022, there was an increase in cases related to infection with the Omicron variant of SARS-CoV-2 [6,8]. A notable characteristic of this variant was the occurrence of milder and less severe symptoms compared to previous variants, especially among vaccinated individuals [9,10]. As this was a retrospective study, the specific variant associated with each case could not be identified, which limited the possibility of more detailed analyses.

Wang and colleagues [10], in a study involving 1139 mild cases of patients infected with the Omicron variant of SARS-CoV-2 in Shanghai, identified cough (57.5%), sputum production (48.3%), and nasal congestion or rhinorrhea (43.4%) as the most frequent clinical manifestations. They concluded that the symptoms caused by Omicron are more complex and should be differentiated from influenza. In our study, the most frequent symptom was also cough, observed in 60% of cases, and when nasal congestion and rhinorrhea were considered together, the prevalence was 48.3%, which is similarly aligned with Wang and colleagues’ findings. A key difference in their study was the analysis based on vaccination status, which was not possible in our investigation due to the lack of systematically recorded vaccination data, representing a limitation of our study.

The present study revealed that fatigue/tiredness was a characteristic COVID-19 symptom, corroborating studies that indicated fatigue/tiredness as one of its main symptoms [2]. The presence of rhinorrhea, ocular symptoms, abdominal pain, rhinosinusopathy, and wheezing/asthma/bronchospasm were characteristic symptoms of the different influenza-like illnesses. Sirijatuphat et al. [5] also observed a difference in rhinorrhea prevalence between COVID-19 and influenza cases, with rhinorrhea being more frequent in the latter. Iyadorai et al. [3] observed that patients with influenza virus infection had a higher prevalence of fever (83.0%) and a similar prevalence of cough compared to patients with COVID-19 infection, whereas other clinical symptoms (sore throat, hoarseness, nasal congestion, rhinorrhea, sneezing, headache, and myalgia) were more common in COVID-19-infected patients. Our results corroborated these earlier findings regarding the sore throat, nasal congestion, headache, myalgia, and cough symptoms, but not the fever symptoms (no difference between the two groups) nor rhinorrhea (greater prevalence in flu-like syndrome cases) [30]. The main discrepancies may arise from differences between the populations in the different studies, and in the SARS-CoV-2 virus variants. At the start of the pandemic, anosmia was considered a predictor of COVID-19 [31]; however, its prevalence decreased with the different variants of the virus [32]. Our study demonstrates the decrease in anosmia prevalence in COVID-19 cases in 2022.

In clinical practice, the identification of factors associated with the disease may be used as guidance to healthcare professionals to conduct targeted investigations. They may also be used as an additional tool where diagnostic resources are limited [5,25]. Moreover, early diagnosis allows for the immediate implementation of isolation measures, contributing to disease transmission control and a decrease of its incidence [11].

Due to the high prevalence of COVID-19 during the study period, a discrepancy between the number of cases and controls was observed. A study conducted in Brazil between January and March 2022, using data from the SIVEP-Gripe system, also demonstrated a large difference in the proportion of hospitalized cases: 99,049 for COVID-19 compared to 4779 for influenza [33].

This study presented some limitations. Approximately 42% of the cases were excluded, 21% for missing data (without reported data on signs and symptoms), which demonstrates a lack of structuring of electronic medical records aimed at research. More structured fields and training for doctors in record filling could decrease the occurrence of missing data, while data imputation strategies could be used for future studies. The control group was limited due to the high prevalence of COVID-19 during the study period; clinical manifestations of COVID-19 may vary according to the viral strain (different variants of SARS-CoV-2) and the vaccination status of the patients (partial or complete vaccination schedule). The use of retrospective data may be affected by information bias, yielding less accurate results.

The use of a database from a telemedicine platform was adequate and sufficient to identify significant factors to differentiate COVID-19 from other influenza-like illnesses for the population studied. Given the context of a pandemic, additional studies should be conducted to identify the similarities between the population served through the telemedicine platform and the local population, in order to enhance the inference and representativeness of the results. It is necessary to improve the acquisition of data through telemedicine by developing more structured electronic medical records. Nevertheless, the use of this data could offer advantages in terms of decreasing costs for studies with a large sample size, ease of conducting studies in adverse situations such as a pandemic, little need for on-site infrastructure, and helping with faster prevention and surveillance actions in the face of worsening conditions.

## 5. Conclusions

Our findings suggest potential predictors associated with influenza-like illness and COVID-19 that may help distinguish between these infections. Fatigue/tiredness and age are associated with COVID-19, whereas the presence of rhinorrhea, ocular symptoms, abdominal pain, rhinosinusopathy, and wheezing/asthma/bronchospasm are associated with influenza-like illness in the population studied. Furthermore, the telemedicine platform analyzed in this study demonstrated significant potential as an important secondary data source. To maximize its utility, it is essential to adopt structured forms with standardized fields and mandatory responses. By structuring and enhancing the usability of such databases, especially during public health emergencies, these platforms can play a critical role in syndromic surveillance, disease prevention, and health promotion strategies.

## Figures and Tables

**Table 1:** Main signs and symptoms observed between COVID-19 and control cases.

	Cases (N = 2419) n (%)	Controls (N = 129) n (%)	*p*-Value
Anosmia/Hyposmia *	71 (2.9)	2 (1.6)	0.584
Cough	1448 (61.5)	80 (62.0)	0.909
Fever	661 (27.3)	39 (30.2)	0.471
Rhinorrhea	654 (27.0)	49 (38.0)	**0.007**
Nasal congestion	515 (21.3)	34 (25.6)	0.248
Sneezing/burning sensation in the nose	107 (4.4)	11 (8.54)	**0.031**
Odynophagia	1027 (42.5)	58 (45.0)	0.575
Myalgia	696 (28.8)	37 (28.7)	0.982
Ocular symptoms *	22 (0.9)	7 (5.4)	**<0.001**
Headache	907 (37.5)	6 (35.7)	0.675
Malaise/Indisposition	295 (12.2)	14 (10.9)	0.649
Dyspnea	112 (4.6)	6 (4.7)	0.991
Diarrhea	158 (6.5)	13 (10.1)	0.117
Chills *	61 (2.5)	4 (3.1)	0.569
Nausea/Sickness	102 (4.2)	7 (5.4)	0.508
Abdominal pain*	43 (1.8)	7 (5.4)	**0.012**
Arthralgia	105 (4.3)	2 (1.6)	0.124
Dizziness *	56 (2.3)	4 (3.1)	0.544
Rhinitis *	8 (0.3)	1 (0.8)	0.374
Asthenia/Adynamia	140 (5.8)	7 (5.4)	0.864
Prostration *	46 (1.9)	1 (0.8)	0.731
Lower back pain*	69 (2.9)	4 (3.1)	0.785
Dermatological symptoms *	12 (0.5)	2 (1.6)	0.156
Fatigue/Tiredness	192 (7.9)	3 (2.3)	**0.019**
Vomiting *	27 (1.1)	2 (1.6)	0.656
Anorexia/Inappetence *	18 (0.7)	4 (3.1)	0.023
Tonsilitis *	4 (0.2)	1 (0.8)	0.229
Rhinosinusopathy *	16 (0.7)	4 (3.1)	**0.016**
Back pain *	46 (1.9)	3 (2.3)	0.736
Chest pain/Palpitation *	69 (2.9)	2 (1.6)	0.582
Wheezing/Asthma/Bronchospasm *	13 (0.5)	4 (3.1)	**0.009**
Insomnia *	1 (0.1)	1 (0.8)	0.099
Aphasia/Dysphonia *	51 (2.1)	4 (3.1)	0.358
Ageusia/Dysgeusia *	49 (2.0)	2 (1.6)	1.000
Expectoration/Secretion *	38 (1.6)	1 (0.8)	0.720
Throat discomfort (irritation, itching, throat clearing, plaques, globules) *	86 (3.6)	1 (0.8)	0.129
Edema *	1 (0.1)	1 (0.8)	0.099
Hearing symptoms (tinnitus, ear fullness, hypoacusis) *	11 (0.5)	2 (1.6)	0.138

* Fisher’s exact test was used in cases with one or more cells with counts less than 5. N/n = absolut number; bold = significant value.

**Table 2:** Multiple binary logistic regression analysis of factors that differentiate COVID-19 from flu syndromes.

Associated Factors	OR	95% CI	*p*-Value
Rhinorrhea	0.572	0.393–0.832	0.003
Ocular symptoms	0.171	0.070–0.415	<0.001
Abdominal pain	0.301	0.129–0.702	0.005
Fatigue	3.308	1.039–10.530	0.043
Rhinosinusopathy	0.186	0.060–0.572	0.003
Wheezing/Asthma/Bronchospasm	0.150	0.047–0.482	0.001
Age	1.016	1.003–1.029	0.015

OR: odds ratio; CI: confidence interval.

## Data Availability

The raw data supporting the conclusions of this article will be made available by the authors on request.

## Supplementary Materials

The following supporting information can be downloaded at: https://www.mdpi.com/article/10.3390/tropicalmed10080213/s1, Figure S1: Flowchart of data selection for the development of a diagnostic prediction model for COVID-19; Table S1: Sex and Age observed between COVID-19 and control cases.

## Abbreviations

The following abbreviations are used in this manuscript:

COVID-19	Coronavirus disease
RT-qPCR	Reverse transcriptase-real time PCR
OR	Odds ratio
CI	Confidence interval
