# Supplementary material for: COVID-19 Clinical Predictors in Patients Treated via a Telemedicine Platform in 2022
Source: Trop Med Infect Dis. 2025 Jul 29;10(8):213. doi: 10.3390/tropicalmed10080213 (PMC12390043; doi:10.3390/tropicalmed10080213)
Supplement: Supplementary file 1 [file tropicalmed-10-00213-s001.zip › tropicalmed-3698788-supplementary.pdf]

Figure S1: Flowchart of data selection for the development of a diagnostic prediction model for COVID-19.

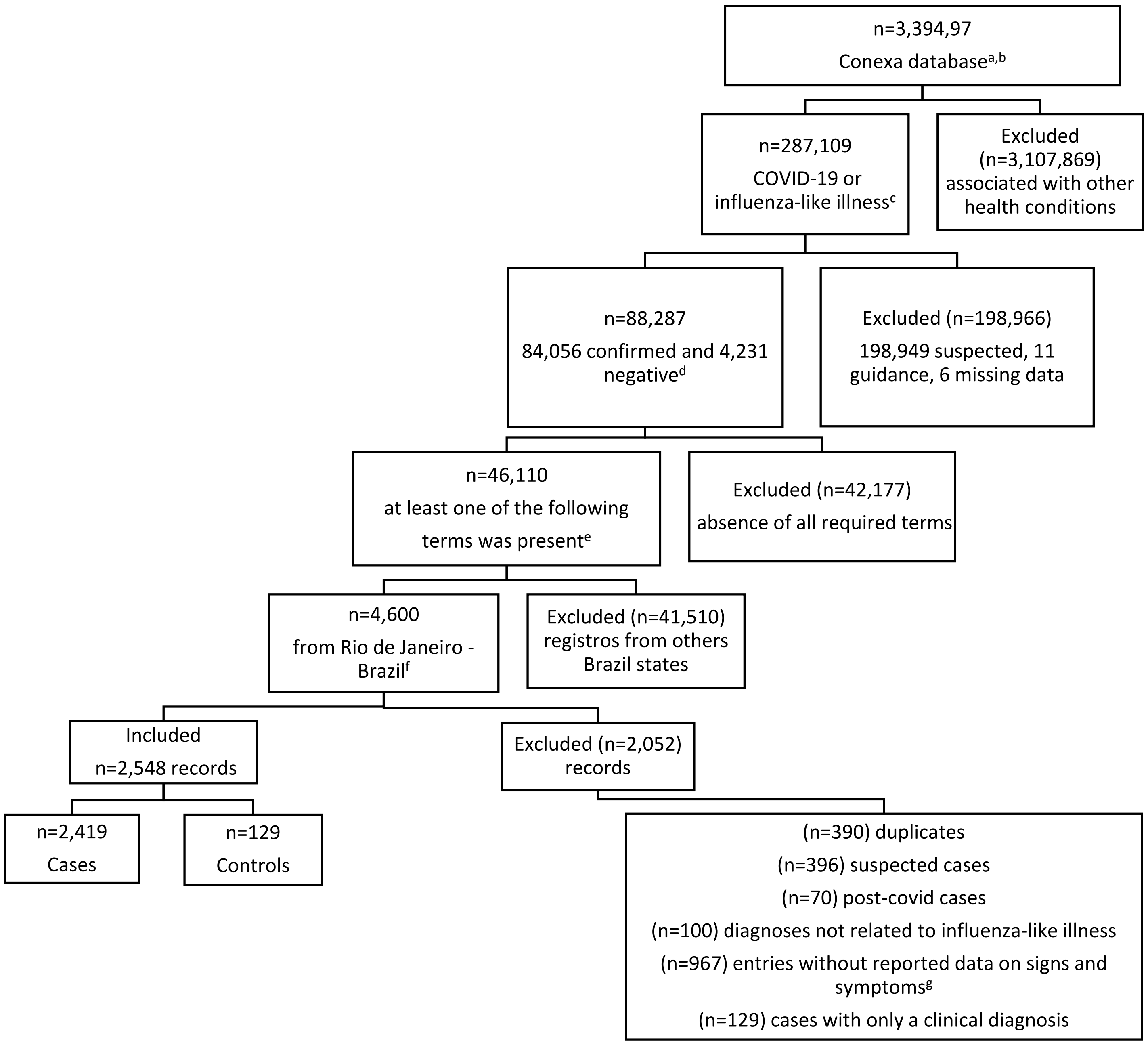

n=3,394,97
Conexa database[a,b]
n=287,109
COVID-19 or influenza-like illness[c]
Excluded (n=3,107,869) associated with other health conditions
n=88,287
84,056 confirmed and 4,231 negative[d]
Excluded (n=198,966)
198,949 suspected, 11 guidance, 6 missing data
n=46,110
at least one of the following terms was present[e]
Excluded (n=42,177)
absence of all required terms
n=4,600
from Rio de Janeiro - Brazil[f]
Excluded (n=41,510) registros from others Brazil states
Included
n=2,548 records
Excluded (n=2,052) records
n=2,419
Cases
n=129
Controls
(n=390) duplicates
(n=396) suspected cases
(n=70) post-covid cases
(n=100) diagnoses not related to influenza-like illness
(n=967) entries without reported data on signs and symptoms[g]
(n=129) cases with only a clinical diagnosis

a. Variables used: *Date of Care; Care Completion Date; Patient ID; Patient State (UF); Epidemic; Epidemic (Medical Report); Sex; Age; Patient Report.*

b. Selection of records related to the topic (COVID-19 or flu-like syndrome) was performed using the variable 'epidemic'.

c. Selection of records classified as confirmed or negative was performed using the variable 'epidemic (medical report)'.

d. Selection using an Excel function [`=IF(ISNUMBER(SEARCH(searched_text; selected_variable)); "1"; "0")`] applied to the variable *'patient report'*. Keywords used in the function: *positive, negative, detectable, detected, confirmed, test, exam, covid, corona*.

e. Selection by place of origin.

f. The variable *'patient report'* was analyzed across all 4,660 records to exclude duplicates, select cases and controls, and identify reported signs and symptoms.

g. Records without specification of signs and symptoms were classified as missing data.

Table S1. Sex and Age observed between COVID-19 and control cases

		Cases (n=2289) n (%)	Controls (n=122) n (%)	Total (n=2411) n (%)	p-value
Sex at birth[a]	Woman	1381 (60.3)	81 (66.4)	1462 (60.6)	
	Man	908 (39.7)	41 (33.36)	949 (39.4)	0,182[c]
		Mean (SD)	Mean (SD)	Mean (SD)	
Age[d]		44.2 (14.8)	40.8 (16.0)	44 (15)	0.008[d]

n: absolute number; %: percentagem value; SD: standard deviation; [a]137 missing data; [b]5 missing data; [c]quiquadrado test; [d]t test
